# Phytosterols from *Dunaliella tertiolecta* Reduce Cell Proliferation in Sheep Fed Flaxseed during Post Partum

**DOI:** 10.3390/md15070216

**Published:** 2017-07-06

**Authors:** Maria Giovanna Ciliberti, Matteo Francavilla, Simona Intini, Marzia Albenzio, Rosaria Marino, Antonella Santillo, Mariangela Caroprese

**Affiliations:** 1 Department of the Sciences of Agriculture, Food and Environment, University of Foggia, Via Napoli, 25-71121 Foggia, Italy; maria.ciliberti@unifg.it (M.G.C.); matteo.francavilla@unifg.it (M.F.); simona.intini@unifg.it (S.I.); marzia.albenzio@unifg.it (M.A.); rosaria.marino@unifg.it (R.M.); antonella.santillo@unifg.it (A.S.); 2 Institute of Marine Science, National Research Council, Arsenale Castello, 2737/F, 30122 Venice, Italy

**Keywords:** cytokines, sheep, post partum, proliferation, phytosterols

## Abstract

The post partum period is characterized by immunosuppression and increased disease susceptibility. Both phytosterols from microalga *Dunaniella tertiolecta* and dietary supplementation with n-3 polyunsaturated fatty acids (PUFA) influence cell proliferation and cytokine release during inflammation. The objective of this paper was the evaluation of the effects of physterols, extracted and purified from *D. tertiolecta*, on the in vitro immune responses of ewes supplemented with flaxseed during post partum. Twenty Comisana parturient ewes were divided in two balanced groups, and supplemented with flaxseed (FS, 250 g/day) or fed with a conventional diet (CON). Blood samples (15 mL) were collected for five weeks, starting from lambing, in order to isolate peripheral blood mononuclear cells (PBMC). Stimulated PBMC were treated with a total sterols fraction from *D. tertiolecta* (TS), a mix of ergosterol and 7-dehydroporiferasterol (purified extract, PE), and a mix of acetylated ergosterol and 7-dehydroporiferasterol (acetylated purified extract, AcPE), extracted and purified from *D. tertiolecta* at two concentrations (0.4 and 0.8 mg/mL). Results of the experiment demonstrated that n-3 PUFA from flaxseed induced an anti-inflammatory cytokine profile, with an increase of both IL-10, IL-6 and a decrease of IL-1β. TS, PE, and AcPE purified from *D. tertiolecta* showed an anti-proliferative effect on sheep PBMC regardless their chemical composition and concentration.

## 1. Introduction

Parturition is considered a physiological acute inflammatory process in which neutrophils and monocytes migrate in the uterus and cervix, followed by an increase in cytokine production, such as interleukins IL-6, IL-1, IL-8 and TNF-α and other chemotaxis factors [1,2]; moreover, it is characterized by a concomitant increase in reactive oxidative species formation [3]. The stressful events of parturition are also accompanied by the production of glucocorticoids (GC)s from the hypothalamus-pituitary-adrenal axis, among which cortisol is the main GC secreted. Circulating cortisol inhibits T-cell proliferation and T-cell development; it has been studied that changes in cortisol levels around parturition are accompanied by immunosuppression, and increasing disease susceptibility [4,5]. The innate immune responses have a predominant role to protect the animal from the development of post partum reproductive diseases [6]. Furthermore, an efficient inflammatory response is required to restore immune homeostasis [7]. The self-regulation of the immune system, consisting in a negative feedback mechanism, includes the secretion of anti-inflammatory cytokines, the inhibition of pro-inflammatory signalling cascades, the changing of receptors for inflammatory mediators, and the activation of regulatory cells [8]. It has been proposed that the intensity and timing of inflammation resolution during post partum could be the crucial factor affecting the pathology of inflammatory disorders [9]. The balance between necessary and excessive aspects of inflammation around parturition is essential to guarantee the recovery of homeostasis, particularly for immune homeostasis; as a result, treatments with nutrients or pharmaceutical agents to avoid disrupting the necessary aspects of inflammatory conditions around parturition have to be explored [9].

Dietary n-3 PUFA are able to change the plasma fatty acid composition and fatty acid profile of phospholipids in the cellular membrane of blood cells such as erythrocytes and PBMC [10,11]. Immune responses, and in particular, a class of lipid mediators called pro-resolving eicosanoids, among which are lipoxins, resolvins and protectins that tends to promote the resolution of inflammation, are influenced by dietary n-3 PUFA [12]. In a study on sheep, pregnancy did not alter mitogen-induced PBMC proliferation and IL-10 production [13]. Whereas, in an vivo sheep study, flaxseed supplementation succeeded to shift cytokine production at two weeks post partum with an increase of both IL-10 and IL-6 production to mitigate excessive inflammation [14].

The microalga *Dunaliella tertiolecta* was proposed by Francavilla et al. [15] for the commercial production of sterols, being a source of phytosterols, among which ergosterol and 7-dehydroporiferasterol are the most abundant. Phytosterols from *D. tertiolecta* were found to play a role on the modulation of PBMC proliferation and on the inflammatory profile activated by cytokines in sheep [16]. An anti-inflammatory action for steroidal compounds has been suggested by the inhibiting action of lipocortin 1, also known as annexin-1 [17].

The aim of the present experiment was the evaluation of the effects of three different extracts of physterols, purified from *D. tertiolecta*, on the in vitro immune responses of ewes supplemented with flaxseed during post partum. In particular, we aimed at investigating: (i) the effects of diet supplementation with flaxseed on sheep PBMC proliferation and cytokine production during post partum; (ii) the effects of in vitro stimulation with a total sterols fraction, a mixture of ergosterol/7-dehydroporiferasterol, and a mixture of acetylated ergosterol/7-dehydroporiferasterol purified from *D. tertiolecta*, on PBMC proliferation and cytokine production of sheep fed flaxseed or not, during post partum.

## 2. Results

### 2.1. Proliferative Response to Phytohemoagglutinin (PHA)

PBMC proliferation was affected by time (*p* < 0.001) and by the interaction time x diet (*p* < 0.001). On average, PBMC proliferation decreased throughout the post partum; in the CON group, PBMC proliferation decreased at 21 days and 28 days compared with parturition; on the contrary, the FS group decreased starting after seven days post partum. At parturition and at 28 days, post partum FS cells proliferated more than CON cells; whereas, after seven days post partum there was a shift, with a reduction of PBMC proliferation in the FS group compared to the CON group (Table 1).

**Figure 1:**
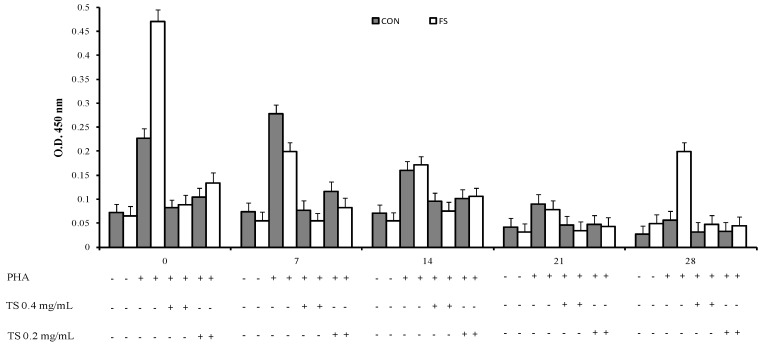
Proliferation of sheep PBMC (Least Squares means ± SEM) from sheep fed flaxseed (FS) or not (CON), and following in vitro stimulation. PBMC were collected from blood samples at 0, 7, 14, 21, and 28 days post partum, and cultured with the total sterol fraction extracted from *Dunaliella tertiolecta* (TS 0.4 mg/mL and 0.2 mg/mL).

**Figure 2:**
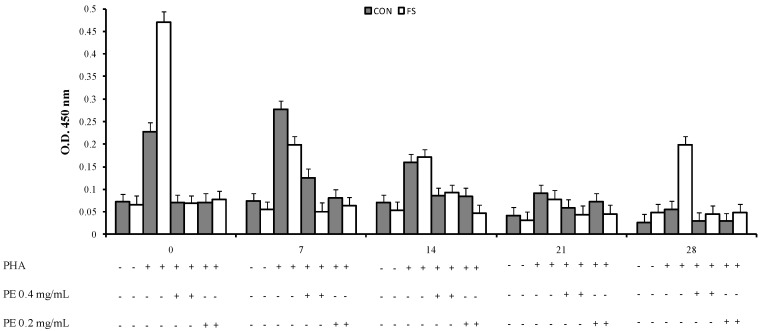
Proliferation of sheep PBMC (Least Squares means ± SEM) from sheep fed flaxseed (FS) or not (CON), and following in vitro stimulation. PBMC were collected from blood samples at 0, 7, 14, 21, and 28 days post partum, and cultured with purified ergosterol and 7-dehydroporiferasterol mixture extracted from *Dunaliella tertiolecta* (PE 0.4 mg/mL and 0.2 mg/mL).

**Figure 3:**
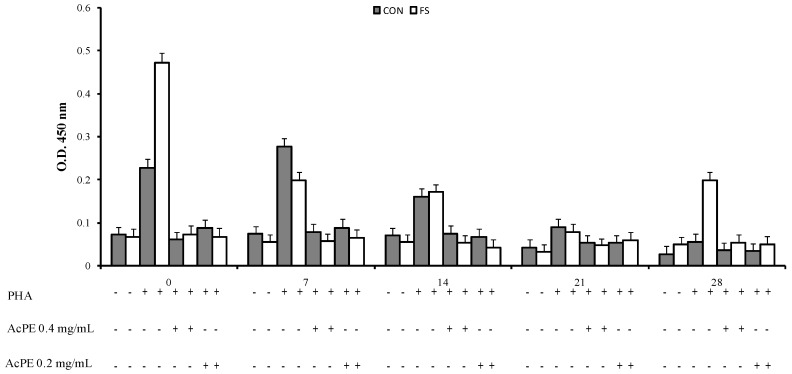
Proliferation of sheep PBMC (Least Squares means ± SEM) from sheep fed flaxseed (FS) or not (CON), and following in vitro stimulation. PBMC were collected from blood samples at 0, 7, 14, 21, and 28 days post partum and cultured with purified acetylated ergosterol and 7-dehydroporiferasterol mixture extracted from *Dunaliella tertiolecta* (AcPE 0.4 mg/mL and 0.2 mg/mL).

An interaction of phytosterol treatment, time and diet (*p* < 0.001, Figure 1, Figure 2 and Figure 3) was registered on PBMC proliferation; the sterols treatment showed a decrease of cell proliferation at parturition and at seven and 14 days post partum in the CON group and at parturition, at seven, 14, and 28 days in the FS group compared to PHA-stimulated cell proliferation.

### 2.2. Cytokine Production by PBMC

**Figure 4:**
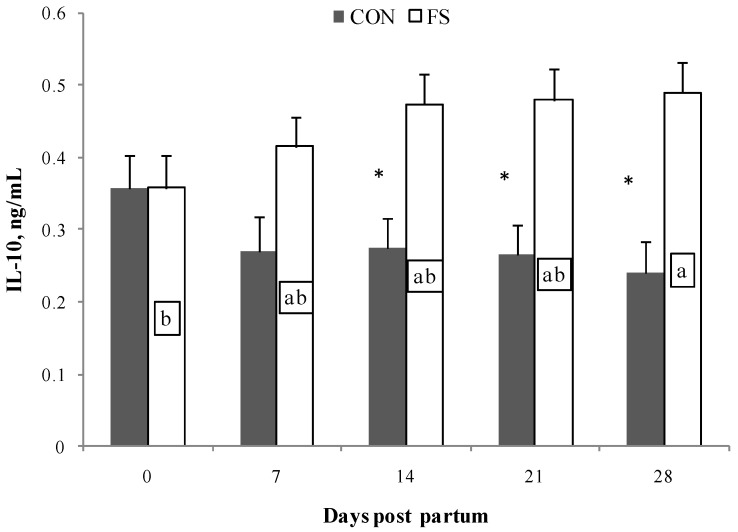
Interleukin (IL)-10 secretion by PBMC (Least Squares means ± SEM) from sheep fed flaxseed (FS) or not (CON), and following in vitro stimulation. PBMC were from blood samples at 0, 7, 14, 21, and 28 days post partum. * indicates significant differences between experimental groups, *p* < 0.05. Different letters indicate significant differences in samples within the same time period, *p* < 0.05.

Cytokine production by PBMC was mainly affected by the flaxseed administration in the diet (*p* < 0.001, Table 2). IL-10 production was affected by diet (*p* < 0.001) and by the interaction of diet×time (*p* < 0.05, Figure 4). At 14, 21, and 28 days, the production of IL-10 by PBMC from the FS group was higher than from CON group; moreover, at 28 days, the FS diet resulted in higher production of IL-10 than at parturition.

**Figure 5:**
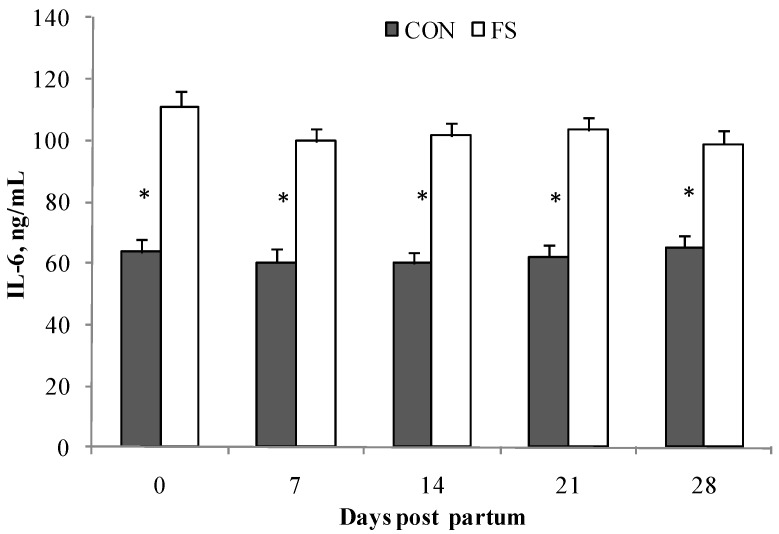
Interleukin (IL)-6 secretion by PBMC (Least Squares means ± SEM) from sheep fed flaxseed (FS) or not (CON), and following in vitro stimulation. PBMC were from blood samples at 0, 7, 14, 21, and 28 days post partum. * indicates significant differences in the same group during time, *p* < 0.05.

The production of IL-6 from PBMC was affected by diet (*p* < 0.001), and by the of interaction diet×time (*p* < 0.05, Figure 5); higher production of IL-6 in the FS group than in the CON group was observed. No effects of stimulation by physterols from *D. tertiolecta* were found.

**Figure 6:**
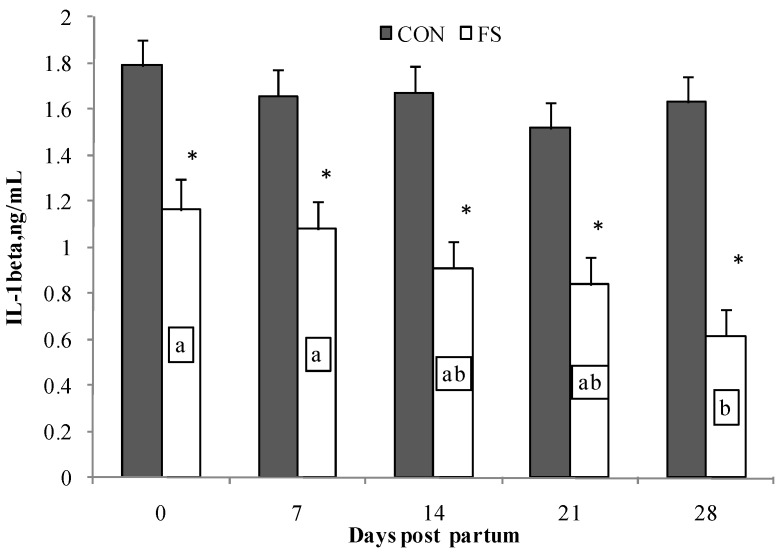
Interleukin (IL)-1β secretion by PBMC (Least Squares means ± SEM) from sheep fed flaxseed (FS) or not (CON), and following in vitro stimulation. PBMC were from blood samples at 0, 7, 14, 21, and 28 days post partum. * indicates significant differences between experimental groups, *p* < 0.05. Different letters indicate significant differences in the same group during time, *p* < 0.05.

IL-1β production by PBMC was affected by diet (*p* < 0.001); PBMC isolated from sheep fed flaxseed registered lower IL-1β production than PBMC isolated from the CON group. Time affected IL-1β production (*p* < 0.05); on average the IL-1β production at seven days post partum was higher than at 28 days post partum in PBMC from the FS group. PBMC from the CON group displayed higher and long lasting production of IL-1β compared with the FS group (Figure 6).

Phytosterols treatment also changed the production of IL-1β (*p* < 0.05); on average, regardless of the diet, both TS and PE at lower concentrations (0.2 mg/mL) resulted in higher IL-1β production than TS and PE at 0.4 mg/mL, and in both stimulated and unstimulated cells, respectively.

## 3. Discussion

This study aimed at evaluating the immunomodulatory and anti-inflammatory activities of a particular mix of phytosterols extracted from the microalga *D. tertiolecta* on sheep PBMC, after in vivo supplementation of sheep diet with flaxseed during post partum.

Disorders of transition periods in animals are associated with dramatic changes in metabolic activity and dysfunctional immune responses [10,18]. When an inflammatory response is in full bloom, a self-limiting and resolving mechanism is activated for limiting excessive tissue damage. The improper balance between infection and self-defence mechanisms leads to post partum reproductive diseases such as puerperal metritis, clinical endometritis, and subclinical endometritis [19,20]. In dairy cows, an increase of energy requirements occurs around parturition [21,22]; as a consequence mechanisms of adaptation emerge, leading to the increase of plasma non-esterified fatty acids (NEFA) and to the alteration of the fatty acid profile of plasma lipid fractions [23], with a decrease in circulating n-3 PUFA [11]. The changes in plasma NEFA composition are the reflection of the fatty acid profile of phospholipids of the cellular membrane of blood cells such as erythrocytes and PBMC [10,11]. Differences in bovine and sheep metabolic disorders during the transition period have not been adequately studied. Results of the present study demonstrated that supplementation with flaxseed, which is rich in n-3 PUFA, influenced PBMC cytokine secretion. Cell culture studies demonstrated that the effects of the n-3 PUFA on inflammatory mediators were related to the alteration of eicosanoid production [8]; n-3 PUFA-derived eicosanoids particularly promote the resolution of inflammation [7]. It is known that circulating n-3 PUFA-derived eicosanoids, such as resolvins, lipoxins, and protectins, up-regulate anti-inflammatory cytokines such as IL-10, to resolve inflammation activated by parturition [9,24]. According to previous studies, the present experiment showed that n-3 PUFA from flaxseed exert an increase in the production of both IL-10 and IL-6 in sheep PMBC. Furthermore, an in vitro study found that pregnancy was not able to influence IFN-γ, IL-4 or IL-10 production by ovine mitogen-activated PBMC [13]. On the contrary, Caroprese et al. [14] observed that flaxseed supplementation in sheep diets succeeded in a shift of cytokine production at two weeks post partum, with an increase of both plasma IL-10 and IL-6 production. The change in secretions of both IL-6 and IL-10 in PBMC from sheep fed flaxseed found in the present experiment, suggested that flaxseed supplementation achieved the modulation of immune responses to overcome immune dysfunction around parturition. Furthermore, it has been stated that the control of inflammatory processes is based on time-dependent IL-6 and IL-10 production; during the early phase of inflammation, IL-6 production can promote inflammatory processes, while in a subsequent phase, IL-6 has the role of limiting inflammation by activating IL-10 production by T-cells [25,26,27]. Accordingly, a time-dependent relationship between IL-6 and IL-10 production from PBMC in the present experiment was observed in sheep fed flaxseed exhibiting an increase IL-10 stimulated by previously high IL-6 levels, two weeks after parturition.

Robinson et al. [28] found that feeding mice a fish oil-rich diet decreased the level of IL-1β mRNA in stimulated spleen lymphocytes, and this reduction was caused by an impaired induction of IL-1 gene transcription. From our results, it emerged that n-3 PUFA from flaxseed caused a reduced secretion of IL-1β, which also resulted in a time-dependent decrease during post partum. Accordingly, at parturition, sheep fed flaxseed showed a decrease of the in vivo levels of IL-1β [14]. The effects of n-3 PUFA on inflammatory cytokine gene expression are exerted by the modification of the activity of nuclear factor kappa-light-chain-enhancer of activated B cells (NF-κB) and/or peroxisome proliferator-activated receptor-γ (PPAR-γ), a member of a family of nuclear receptors named PPARs; moreover, IL-6 is able to inhibit IL-1β expression [29].

Results from the present study demonstrated the main role exerted by flaxseed in the diet of sheep in driving cytokine production with respect to phytosterols from stimulated PBMC. In a previous study [16], when ovine PBMC were cultured in the presence of a mixture of ergosterol and 7-dehydroporiferasterol extracted and purified from *D. tertiolecta*, the levels of IL-6 and TNF-α decreased, whereas the IL-10 level increased, confirming a potential anti-inflammatory effect of phytosterols. The absence of the direct effect of sterols on cytokine production in the present experiment could be ascribed to the simultaneous actions exerted on PBMC by phytosterols and n-3 PUFA from flaxseed in the diet, and to the differences in the mechanism of action of phytosterols as compared with n-3 PUFA on immune responses. Several studies demonstrated that n-3 PUFA in the diet can alter eicosanoid production from PUFA incorporated into the membrane phospholipids of immune cells, and, as a consequence, cytokine production [7].

Very little information is available regarding the alteration of mitogen-induced PBMC proliferation during ovine pregnancy [13,30]. Wattegedera et al. [13] did not find differences on average between mitogen-induced PBMC proliferation of pregnant and non-pregnant sheep. Steroidal compounds are known as potent anti-inflammatory substances, and their anti-inflammatory mechanism of action was described by Vane and Botting [17]. Results from the present experiment on PBMC proliferation demonstrated that sterols from *D. tertiolecta* during post partum exerted an anti-proliferative response on PBMC. Immunological activities of phytosterols have been widely studied [14,31]; their effects on cell proliferation are usually associated with their structural features [32]. In particular, phytosterols such as β-sitosterol-β-d-glucoside and its aglycone showed anti-ulcerative activities in rats affected by chronic gastric ulcer [33]. Whereas, ergosterol and ergosterol peroxide demonstrated inhibitory effects on induced inflammation in mouse skin [34], and on RAW264.7 mouse macrophage-like cells [35]. Accordingly, in a previous experiment it was found that the purified *D. tertiolecta* extract, consisting of a mixture of 7-dehydroporiferasterol and ergosterol at 0.8 mg/mL, was able to suppress cell proliferation of sheep ConA-stimulated PBMC [16]. In the present experiment all the sterols tested exerted an anti-proliferative effect on PBMC apart from their molecular structures (acetylated or not, and mixtures or purified sterols) and apart from the concentrations tested. In particular, the anti-proliferative effect was evident around parturition until the second week after parturition. Recently, suppressed proliferation and tumorigenicity of ovarian carcinoma cells was induced by the inhibition of phospholipase A2 activity [36]. Steroidal compounds are able to suppress phospholipase A2 activity by the synthesis of lipocortin-1, thus blocking eicosanoid production from arachidonic acid [17]. As a consequence, taking our results into account, the antiproliferative effect of phytosterols from *D. tertiolecta* on PBMC could be attributed to their ability to inhibit eicosanoid production from arachidonic acid by suppressing phospholipase A2 activity. Further studies are needed to confirm previous hypothesis and the mechanisms of action of phytosterols from *D. tertiolecta* on phospholipase A2 activity. Furthermore, results from the present study corroborated previous data on the antiproliferative effects of extracts from *D. tertiolecta* on sheep PBMC, and suggest their utility into controlling tissue damage caused by excessive inflammatory reactions.

## 4. Materials and Methods

### 4.1. Extraction and Purification of Total Sterols Fraction, of Ergosterol/7-Dehydroporiferasterol, and of Acetylated Ergosterol/7-Dehydroporiferasterol Mixture from D. tertiolecta

Total sterols were extracted and separated from *D. tertiolecta* as described by Francavilla et al. [37]. Briefly, total lipids extracted according to Bligh and Dyer [38] from freeze dried biomass, were saponified by refluxing in a 5% (*w*/*v*) KOH methanol:water (4:1. *v*/*v*) solution for 2 h. The unsaponified fraction (containing sterols not esterified) was separated and concentrated. Total sterols from unsaponified material were purified by flash liquid chromatography (FLC). A column for FLC (h 70 cm × Ø 2 cm, Sigma Aldrich, Milan, Italy) was packed with activated silica gel (0.04–0.063 mm, 230/400 mesh ASTM, Carlo Erba Reagents, Milan, Italy). It was eluted isocratically with hexane:ethyl acetate 8:2 (*v*/*v*) and with a loading ratio of unsaponified-to-stationary phase of 2% (*w*/*w*), and N_2_ pressure was set at 2 bar with a corresponding elution flux of 4 mL min^−1^.

Silver ion flash chromatography (Ag-FLC) was used for purifying ergosterol (22*E*.24*R*)-methylcholesta-5.7.22-trien-3β-ol) and 7-dehydroporiferasterol ((22*E*.24*R*)-ethylcholesta-5.7.22-trien-3β-ol). The activated Ag-silica gel was used as the stationary phase in the previously described column for FLC. The column was eluted in isocratic conditions with hexane-ethyl acetate 8:2 (*v*/*v*), a loading ratio of 1% (*w*/*w*), and a pressure of N_2_ set at 2 bar with a corresponding elution flux of 4 mL/min. Fractions containing only ergosterol and 7-dehydroporiferasperol were combined, and the solvent was removed on a rotary evaporator (Büchi Rotavapor, Flawil, Switzerland).

Acetylation of ergosterol and 7-dehydroporiferasperol was achieved to increase their lipophilicity using acetic anhydride (Ac_2_O) and anhydrous NiCl_2_ as a catalyst under solvent free-conditions according to the method reported by Meshram and Patil [39]. The mixture of sterols (2 mmol), acetic anhydride (4 mmol), and 0.1 mol% anhydrous nickel chloride was stirred magnetically at room temperature for 2 h. The reaction mixture was diluted with water (30 mL) and extracted with chloroform. The combined chloroform extracts were washed successively with 10% sodium bicarbonate and water, dried with Na_2_SO_4_, and concentrated under reduced pressure. Conversion rates and reaction yields were higher than 95%.

### 4.2. Analyses by Gas Chromatography-Mass (Tandem) Spectrometry

**Figure 7:**
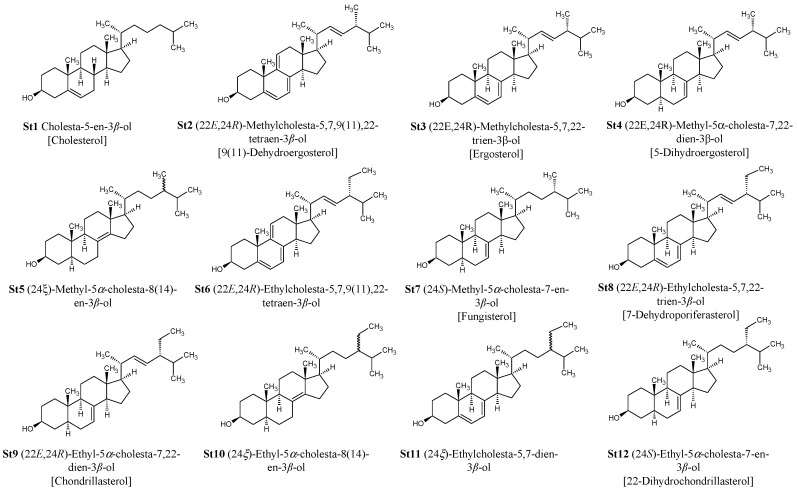
Molecular structures of sterols purified from *Dunaliella tertiolecta*.

**Figure 8:**
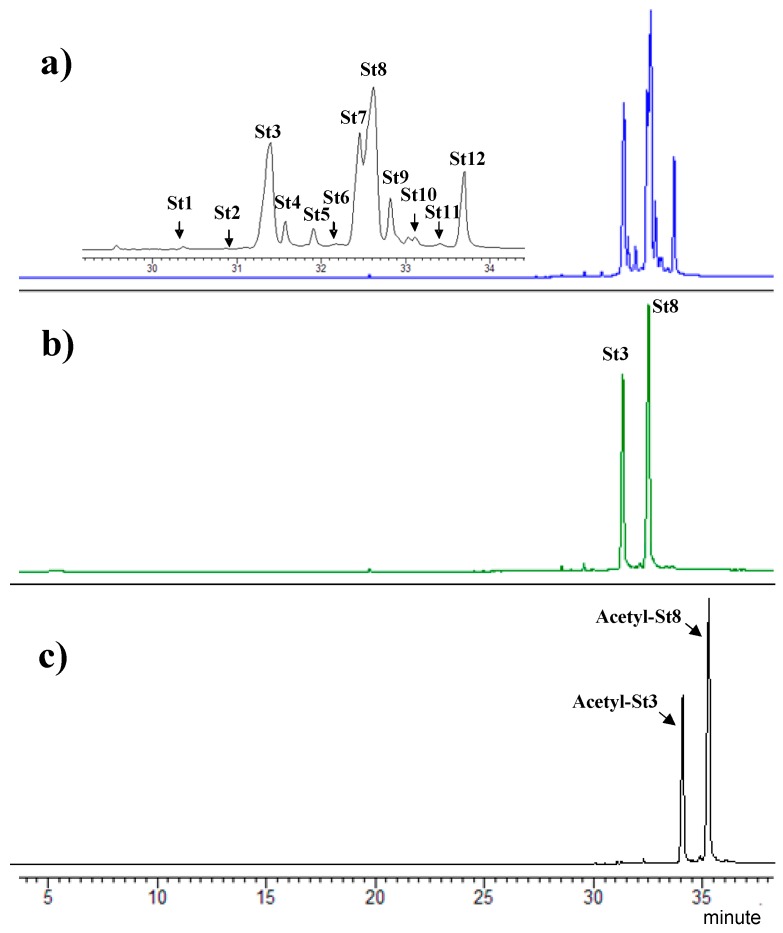
Total Ion Chromatograms of the totals sterols fraction (**a**), purified ergosterol and 7-dehydroporiferasterol mixture (**b**), and acetylated ergosterol and 7-dehydroporiferasterol mixture (**c**) extracted from *Dunaliella tertiolecta.*

Purified and acetylated sterols from *D. tertiolecta* were characterized by gas chromatography-mass (tandem) spectrometry GC-MS/MS analysis (GC-MS/MS Ion Trap 240, Agilent Technologies, Santa Clara, CA, USA). A VF-5 ms (Agilent J&W, Santa Clara, CA, USA) capillary column (30 m × 0.25 mm × 0.25 μm film thickness) was used for the analyses. The GC carrier gas was He (1.0 mL/min). The injector temperature was 250 °C. The temperature program was as follows: constant temperature of 50 °C for 1 min followed by the first temperature ramp to 300 °C at 5 °C min^−1^, and finally a constant temperature of 300 °C for 10 min; then a second temperature ramp to 325 °C at 10 °C min^−1^ holding for 1.5 min. The ion trap was held at 230 °C, the manifold at 80 °C, and the transfer line was 250 °C. The GC-MS was operated in electron ionization (EI) and chemical ionization (CI) mode over a mass range of 50–800 *m*/*z*. The chemical ionization mode was used to confirm the molecular weight (M + 1) of compounds. Identification of sterols was based on the comparison of their retention times relative to authentic standards, mass spectra of authentic standards, and available spectra in NIST05 and Wiley 07 mass spectral libraries. Sterol standards used for identification include cholesterol (C_27_ ∆^5^) and ergosterol (C_28_ ∆^5,7,22^). The identification of fungisterol (C_28_ ∆^7^) and 7-dehydroporiferasterol (C_29_ ∆^5,7,22^) was established based on mass spectra described in the literature [40,41,42]. The molecular structures of 12 sterols extracted from *D. tertiolecta* are reported in Figure 7, while total ion chromatograms (GC-MS) of purified and acetylated sterols are reported in Figure 8.

### 4.3. Animals and Experimental Treatments

Twenty parturient Comisana ewes were randomly selected from an intensively managed flock of Segezia research station of the Council for Research and Experimentation in Agriculture (CRA-ZOE). Apulia (latitude: 41°27’6″ and longitude: 15°33’5″). The ewes involved in the experiment were randomly divided in two groups of 10, balanced for age, parity, body weight, and body condition score. Ewes were healthy and their conditions were judged as good at the commencement of the trial. The ewes were carefully examined by veterinarians to exclude the presence of signs of clinical mastitis (pain, gland swelling, fever) and a small quantity of milk was checked visually for signs of mastitis. No cases of mastitis were detected during the study period.

All groups were individually fed twice daily and received 1.8 kg/ewe/day of oat hay. The control group (CON) received 1 kg/day of pelleted concentrate (Mangimificio Molino Gallo, Potenza, Italy); the flaxseed group (FS) was fed a supplementation of whole flaxseed (Lin Tech, Tecnozoo srl, Torreselle di Piombino Dese, Italy), receiving 750 g/ewe/day of pelleted concentrate, and 250 g/ewe/day of whole flaxseed. Animals both in CON and FS groups were fed the experimental diet starting from one week before lambing.

The chemical composition of diets was determined with standard procedures [43]. The determination of methyl esters in the diet ingredients was carried out according to O’Fallon et al. [44]. The concentrations of fatty acid methyl esters (FAME)s were determined utilizing a calibration curve with a mixture of standards of 50 fatty acid (GLC Reference standard 674, Nu-Check Prep, Inc., Elysian, MN 56028, USA) with added CLA standards: C18:2-8t, 10c; C18:2-9c, 11t; C18:2-11c, 13t; C18:2-9t, 11c; C19:2-8c, 10c; C18:2 9c, 11c; C18:2-10t, 12c; C18:2-8t, 10t; C18:2-9t, 11t; C18:2-10t, 12t; C18:2-11t, 13t (GLC Reference standard UC-59M, Nu-Check Prep. Inc., Elysian, MN 56028, USA). The fatty acid composition of the ingredients of the diet are reported in Table 3.

### 4.4. Isolation of PBMC

Blood samples (15 mL) were collected in vacuum tubes from the jugular veins of the sheep. Isolation of PBMC was performed by density gradient centrifugation according to Wattegedera et al. [45]. Peripheral blood mononuclear cells were resuspended at a final concentration of 2 × 10^5^ cells/mL in Iscove’s Modified Dulbecco’s medium (IMDM) (Sigma Aldrich, Milan, Italy) supplemented with 10% fetal bovine serum(FBS) and 50 μg/mL gentamicin.

### 4.5. PBMC for Lymphocyte Stimulation Assay and Cytokine Determination

Lymphocyte proliferation assays were performed by adding 100 μL of cell suspension into quadruplicate wells of 96 well U-bottom plates. PBMC were treated with a total sterols fraction (TS), and a mix of ergosterol and 7-dehydroporiferasterol extracted and purified from *D. tertiolecta* (Purified Extract, PE), and a mix of acetylated ergosterol and 7-dehydroporiferasterol were extracted and purified from *D. tertiolecta* [acetylated purified extract, (AcPE)], and 50 μL of PHA (Sigma-Aldrich, Milan, Italy) at a final concentration of 5 μg/mL. For each treatment, 0.0 mg/mL, 0.4 mg/mL and 0.8 mg/mL were tested on PBMC to verify the effects of sterols on their proliferation. Negative control wells contained 100 μL of PBMC suspensions without mitogen (Negative Control, NC). Positive control wells contained 100 μL of PBMC suspensions with PHA (PHA-Stimulated Cells, SC). The plates were incubated at 37 °C and 5% CO_2_ in a humidified incubator for 96 h. After 96 h of incubation, cell suspensions were centrifuged at 1000 *g* for 5 min, and cell-free supernatants from each well were collected and stored at −20 °C for ELISA testing to measure cytokine production.

In order to test lymphocyte proliferation after 96 h of incubation, a 5-Bromo-2′-Deoxyuridine (BrDU) test was performed using a commercial kit (Exalpha Biologicals Inc., Shirley, MA, USA). After 18 h of incubation, BrDU incorporation during DNA synthesis was measured by reading the optical density with a titer-ELISA spectrophotometer (Power Wave XS, Biotek, Swindon, UK) at 450 nm.

### 4.6. Determination of Interleukins in Culture Supernatant by ELISA Testing

Assays were optimized for concentrations of mouse monoclonal antibodies (mAb), supernatants, polyclonal detecting Ab and secondary conjugate Ab. The levels of IL-6 and the IL-1β in cell-free supernatants were determined by capture ELISA performed in 96-well microtiter plates, according to Caroprese et al. [46] with some modifications. Optical density was measured at a wavelength of 450 nm. Samples were read against a standard curve obtained using a scalar dilution of *r* bovine IL-6, and IL-1β (Kingfisher Biotech Inc., St. Paul, MN, USA). Data were expressed as ng/mL of both IL-6 and IL-1β.

The determination of IL-10 in supernatants was assayed by an ELISA test according to Kwong et al. [47] and Hope et al. [48] with some modifications; 96-well plates (Sterilin, Newport, UK) were coated overnight at 4 °C with 100 μL of mAb anti-Bovine IL-10 (Serotec Ltd., Kidlington, UK, 2 μg/mL in PBS). All the incubations were performed at room temperature. The levels of IL-10 were measured colorimetrically at 450 nm and quantified by interpolation from the standard curve. The ELISA for IL-10 detection was standardized using biologically active recombinant bovine IL-10 (Kinfisher Biotech Inc., St. Paul, MN, USA). Data were expressed as ng/mL of IL-10.

All procedures were conducted according to the guidelines of the European Union (EU) Directive 2010/63/EU [49] on the protection of animals used for experimental and other scientific purposes.

### 4.7. Statistical Analysis

All variables were tested for normality using the Shapiro-Wilk test [50]. Then, data were processed using Analysis of Variance (ANOVA) with the REPEATED statement in PROC MIXED with variance components (CV) as the covariance structure of Statistical Analysis Software (SAS) [51]. Diet (DT), time of sampling (TM), phytosterols treatment (PS), and their interactions were fixed factors. Animal was a random factor nested in the treatment. Significant differences between means were found (*p* < 0.05).

## 5. Conclusions

The findings of our study demonstrated that the supplementation with flaxseed, rich in n-3 PUFA, affected the immune responses of sheep during post partum. Dietary n-3 PUFA induced an anti-inflammatory cytokine profile, with an increase of IL-10 and IL-6, and a decrease of IL-1β.

The in vitro stimulation of PBMC with a total phytosterols fraction, a mixture of ergosterol/7-dehydroporiferasterol, or a mixture of acetylated ergosterol/7-dehydroporiferasterol purified from *D. tertiolecta*, showed an anti-proliferative effect. These results underlined the possibility for utilizing total sterols and a mix of 7-dehydroporiferasterol and ergosterol purified and extracted from *D. tertiolecta* during post partum, and also at lower concentration, as a potential anti-inflammatory natural product. This event could be of particular interest for protecting tissues from damage caused by excessive inflammatory responses in sheep during post partum. Further investigations are needed to identify the mechanisms of action for the anti-proliferative properties of sterols from *D. tertiolecta*.

## Figures and Tables

**Table 1:** Proliferation of sheep peripheral blood mononuclear cells (PBMC) (Least Squares means ± SEM) supplemented or not (CON) with flaxseed (FS), during the post partum period.

Diet	Days Post Partum				
	0	7	14	21	28
CON	0.097 ± 0.006 Ab	0.115 ± 0.007 Aa	0.092 ± 0.006 A	0.058 ± 0.007 B	0.034 ± 0.007 Cb
FS	0.130 ± 0.008 Aa	0.078 ± 0.007 Bb	0.080 ± 0.006 B	0.048 ± 0.007 C	0.067 ± 0.007 ABa

A,B,C Means followed by different capital letters are significantly different among time of sampling at *p* < 0.05. a,b Means followed by different letters are significantly different among diet at *p* < 0.05.

**Table 2:** IL-10, IL-6 and IL-1 β concentration (Least Squares means ± SEM) in PBMC from sheep during post partum period, supplemented or not (CON) with flaxseed (FS), and cultured in the presence of total sterols (TS), purified extract (PE), and acetylated purified extract (AcPE) extracted and purified from *Dunaliella tertiolecta*.

Cytokines	Diet	PBMC Treatment								SEM	Effect, *p*		
		NC	SC	TS	TS	PE	PE	AcPE	AcPE				
				0.2 mg/mL	0.4 mg/mL	0.2 mg/mL	0.4 mg/mL	0.2 mg/mL	0.4 mg/mL		DT	TM	PS
IL-10	CON	0.347	0.322	0.277	0.314	0.238	0.233	0.256	0.266				
	FS	0.439	0.473	0.468	0.568	0.492	0.401	0.348	0.353	0.06	***	NS	NS
IL-6	CON	64.498	58.967	59.832	63.179	62.576	60.681	62.050	66.274				
	FS	99.183	101.890	102.170	101.170	105.610	103.940	105.120	104.900	5.23	***	NS	NS
IL-1β	CON	1.462	1.443	1.995	1.498	2.034	1.449	1.747	1.614				
	FS	0.761	0.873	1.059	0.945	0.927	0.891	1.008	0.920	0.15	***	*	*

NC = Negative Control, SC = PHA-Stimulated Cells, DT = Effect of diet, TM = Effect of time of sampling, PS = Effect of phytosterols. *** *p* < 0.001, * *p* < 0.05.

**Table 3:** Fatty acids composition of the concentrate, oat hay and flaxseed.

Fatty Acids, g/100 g of Total Fatty Acids	Concentrate ^1^	Oat Hay	Flaxseed ^2^
C14:0	16.09	5.62	29.04
C16:0	2.64	3.67	5.41
C18:0	19.05	19.50	20.85
C18:1cis-9	53.73	16.15	20.84
C18:2cis-9cis-12	6.87	54.03	11.79
C18:3n3	1.77	1.18	12.07

^1^ Contained: Corn Meal, Soybean Meal, Wheat Germ Meal, Wheat Meal, Roasted Soybean Seeds, Barley Meal, Wheat Fine Bran, Corn Cracked, Sugarcane Molasses, Partially Debarked Sunflower meal, Bentonite, Dried Pulp, Calcium Carbonate, Sodium Bicarbonate, Sodium Chloride, Magnesium Oxide, 8.3 IU/g vitamin A, 8.2 IU/g vitamin D3, 99 mg/kg vitamin E, 0.07 mg/kg vitamin B1, 255 mg/kg vitamin PP, 488 mg/kg Cl, 293 mg/kg Fe, 1.26 mg/kg Co, 1 mg/kg Cu, 0.4% Na. ^2^ Lin Tech (Tecnozoo srl, Torrreselle di Piombino Dese, Italy).
